# Flavanol-Rich Cacao Mucilage Juice Enhances Recovery of Power but Not Strength from Intensive Exercise in Healthy, Young Men

**DOI:** 10.3390/sports6040159

**Published:** 2018-11-28

**Authors:** Paul T. Morgan, Paola M. Wollman, Sarah R. Jackman, Joanna L. Bowtell

**Affiliations:** Department of Sport and Health Sciences, College of Life and Environmental Sciences, University of Exeter, St. Luke’s Campus, Exeter EX1 2LU, UK; p.t.morgan@exeter.ac.uk (P.T.M.); pmw216@exeter.ac.uk (P.M.W.); S.Jackman@exeter.ac.uk (S.R.J.)

**Keywords:** anti-inflammatory, anti-oxidant, cacao juice, flavanols, muscle damage, recovery

## Abstract

(1) Background: Mucilage within cacao pods contains high levels of polyphenols. We investigated whether consumption of cacao juice enhances the recovery of muscle function following intensive knee extension exercise. (2) Methods: Ten recreationally active males completed two trials of 10 sets of 10 single leg knee extensions at ~80% one repetition maximum. Participants consumed each supplement (ZumoCacao^®^ juice, CJ or a dextrose drink, PL) for 7 days prior to and 48 h post exercise. Knee extension maximum voluntary contraction (MVC) and a counter movement jump (CMJ) were performed at baseline, immediately, 24 h, and 48 h post-exercise. Venous blood samples were collected at each time point and analyzed for indices of inflammation, oxidative damage, and muscle damage. (3) Results: CMJ height recovered faster with CJ at 24 h and 48 h post-exercise (*p* < 0.05), but there was no effect of CJ on recovery of MVC (both *p* > 0.05). There was also no effect of the trial on any blood markers (all *p* > 0.05). (4) Conclusions: Supplementation with CJ for 7 days prior to and 2 days after intensive knee extensor exercise improved functional recovery as shown by an improved recovery of CMJ up to 48 h post-exercise. However, the precise mechanism of action is unclear and requires further investigation.

## 1. Introduction

The use of nutritional supplements is widespread among both elite athletes and recreational sportsmen and women as a means to enhance performance, support rapid recovery between training sessions, and maximize the improvements in performance that are achieved via training [1]. Novel or unaccustomed exercise results in extensive muscle damage with symptoms of muscle soreness and impaired muscle force production persisting for up to two to five days [2,3]. It is likely that the decreased muscle force generating capacity is due to myofibrillar disruption and structural damage to the muscle as shown by the increase in the blood concentration of large intracellular muscle proteins such as creatine kinase (CK) as well as impaired excitation-contraction coupling related to altered intracellular calcium homeostasis and increased muscle protein degradation [3,4,5,6].

The initial, primary, phase of damage is suggested to occur as a consequence of both the mechanical forces to which the muscle fibers are exposed and oxidative stress due to exercise-induced increases in reactive oxygen species (ROS) and nitric oxide (NO) derivatives that exceed the antioxidant defense capacity [7]. A second phase of damage occurs due to the inflammatory response to muscle injury [7]. As a consequence of the apparent role for ROS and NO derivatives in muscle damage, there has been considerable interest in the efficacy of polyphenol supplementation in reducing the symptoms associated with muscle damage due to their anti-oxidant and anti-inflammatory properties [8].

We and others have shown that polyphenol-rich Montmorency cherry juice enhances the recovery of muscle function after intensive exercise through reduced oxidative damage and inflammation [9,10,11] due to their high concentrations of phytochemicals such as anthocyanins [9,10,11,12,13,14,15]. In addition, blueberry (anthocyanins [16]), green tea (catechins [17]), and pomegranate (epigallocatechins [18,19]) have been found to support more rapid recovery. Recently, Hutchison et al. (2016) also demonstrated the effects of blackcurrant nectar supplementation four days prior to and four days post a bout of eccentric knee extension exercise to reduce muscle damage and inflammation compared to placebo via a reduction in CK levels at both 48 and 96 h post exercise and interleukin 6 (IL-6) 24 h post exercise. However, no measure of performance or functional recovery was included [20].

Cacao beans used for cocoa production (conventionally known as ‘Theobroma cacao’) are abundant in flavanols and proanthocyanidins [21,22,23,24,25]. The potential antioxidant and anti-inflammatory qualities of cacao polyphenols [26] suggest that improvements in recovery could also be achieved with cacao supplementation. To date, only one study has tested this hypothesis [27] and found that recovery from downhill running was not enhanced after cacao supplementation. However, the downhill running protocol did not result in any impairment in a 5 km time trial performance or knee extensor isometric force in the participants who were trained runners. In such circumstances, it was not possible to demonstrate improved recovery after cacao supplementation. Consumption of chocolate milk has been shown to improve recovery relative to a carbohydrate placebo, but it is likely that the protein component of the chocolate milk contributes to this attenuation in muscle damage since the flavanol and epicatechin levels in the chocolate milk are unlikely to be sufficiently high [28]. 

Cacao juice (CJ) is a novel product derived from the mucilage of the cacao pod that surrounds the beans, which was previously discarded or utilized for animal feed. To date, we are unaware of any research that has attempted to investigate the potential of CJ to favorably enhance recovery from intense exercise. Therefore, this is the first study to investigate the effects of CJ on recovery from intensive exercise, which possesses a blend of polyphenols alongside a high dose of carbohydrate. We hypothesized that the CJ polyphenols will reduce oxidative modification of proteins and reduce inflammation and, thus, enhance recovery from muscle damage. We, therefore, investigated whether, in comparison to a carbohydrate-matched placebo, consumption of CJ improved functional recovery from intensive exercise in healthy, young men.

## 2. Materials and Methods

### 2.1. Participants

Ten healthy recreationally active male volunteers (mean ± SD: age 22.8 ± 3.3 years, height 1.84 ± 0.59 m, body mass 85.3 ± 12.0 kg, single leg knee extension one repetition maximum, 1RM: 90.4 ± 19.0 kg) provided written, informed consent to participate in the present study, which was approved by the University of Exeter Sport and Health Sciences ethics committee. All participants reported completing 3–4 resistance training sessions per week. Participants were instructed to arrive at the laboratory on the day of ‘muscle damaging’ exercise in a rested and fully hydrated state, following an overnight fast, to avoid consumption of caffeine and alcohol in the 24 h prior to each testing session. Participants were also instructed to refrain from strenuous physical activity for 48 h prior to and following the intensive exercise protocol (48 h post). Participants recorded their diet for 48 h prior to and 24 h post the single leg intense knee extension exercise protocol (as described below) and then replicated this diet for the second, cross-over, trial. In the absence of published data on the effects of CJ, sample size calculations were based on the Montmorency cherry supplementation effect size [10]. With 80% power, 5% significance, and an effect size of 1.0 for recovery of MVC, 10 participants are required.

### 2.2. Familiarization

Familiarization testing was conducted 1–2 days prior to day 1 of supplementation. Participants were fully familiarized with all experimental procedures including completing single leg, knee extension maximum voluntary isometric contractions (MVC). During this familiarization visit, the single leg knee extension 1RM was also determined for each leg by performing a standard ramp test [29]. These data were required to set the work load for the subsequent intense knee extension exercise protocol. Participants were seated on the knee extension machine (Leg Extension,TechnoGym UK Ltd., Cesena, Italy) with each weight lift assessed by the investigator and considered successful if performed with proper technique within the metronome-guided time interval (2 s) and going through the full range of motion (0.7 rad). Following the measurement of 1RM, participants completed three separate counter movement jumps (CMJ) and a muscle soreness visual analogue scale (VAS) to familiarize them with the experimental protocol as described below. The typical CMJ test (i.e., dual leg) was selected during pilot testing since participants found it difficult to balance on one leg following the intense knee extension exercise protocol.

### 2.3. Experimental Design

**Figure 1:**
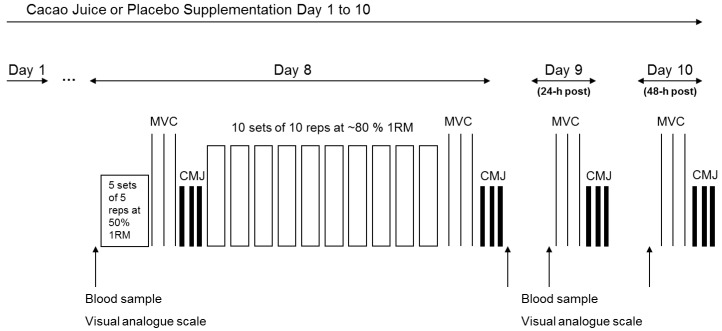
Schematic overview of the experimental protocol.

Participants completed two trials separated by a minimum two-week wash-out period in a double-blind, randomized, counter-balanced, cross-over fashion in order to reduce bias via leg dominance and familiarization (Figure 1). Participants consumed 330 mL once per day for 10 days of either Ecuadorian cacao juice (ZumoCacao^®^, CJ) or a carbohydrate and flavor-matched placebo (PL). For each supplementation period, participants completed the single leg exercise on day 8 of supplementation with different legs used for each trial in order to minimize any repeated bout effect of eccentric exercise and subsequent muscle damage [30,31,32]. 

Experimental visits commenced at ~8:00 a.m. in order to account for diurnal variation. On arrival to the laboratory (day 8), 10 mL resting baseline blood samples were taken from the antecubital vein. A VAS was used as an index of muscle soreness in the lower limbs using a 100 mm scale with ‘No soreness’ at one end and ‘As sore as it could possibly be’ at the other. On each occasion the VAS was used, participants were asked to sit on a standardized chair and rate their soreness on the scale based upon the soreness associated to this task. Participants completed a standardized warm-up at 50% 1RM prior to completing 3 × 3 s single leg MVCs at 60° of knee flexion (verified by goniometer) separated by 60 s rest using an isokinetic dynamometer (Biodex System 3, Shirley, NY, USA) for assessment of quadriceps muscle strength. Participants were given standardized verbal encouragement for the duration of each 3 s contraction. Participants then completed 3 × CMJ separated by a 60-s rest period using a standardized protocol. The participants were asked to stand on the jump mat (Probotics Inc. 8602 Esslinger CT, Huntsville, AL, USA) with their feet parallel and approximately shoulder-width apart. Participants then completed a maximal vertical jump while maintaining hands on hips throughout. Using this experimental set up, pilot testing revealed CMJ height and MVC force to have a coefficient of variation (CV%) within our lab of 1.4% and 2.9%, respectively. 

Following 5 min rest, participants completed a single leg exercise protocol designed to induce knee extensor muscle damage, which was comprised of 10 sets of 10 repetitions at 80% concentric 1RM with a short (<2 s) concentric and elongated eccentric phase (3 s). Each set was separated by a 2-min rest period. If participants were unable to maintain the workload, the load was decreased by 10% and this was then matched during the second trial. The % 1RM during the intense knee extension exercise protocol was then retrospectively calculated. Water was provided for participants to drink ad libitum. After completing the 10 sets, participants were asked to repeat the baseline measures (i.e., MVC, CMJ, VAS, and blood withdrawal). Participants then arrived at the laboratory again at 24 h and 48 h post exercise to repeat the previously mentioned measurements for assessment of recovery from muscle damage. 

Participants were instructed to take one 330 mL serving of their supplement (described below) each morning. However, on day 8 (i.e., the day on which intense knee extension exercise protocol was performed), participants were advised to consume the juice immediately following the intense knee extension exercise protocol. In addition, 24-h and 48-h after the intense knee extension exercise protocol (i.e., day 9 and 10), participants ingested the supplement 60 min before arrival to the laboratory. Previous research has demonstrated that plasma anthocyanin bioavailability increases to a peak between 1–2 h post ingestion [33,34,35], which coincides with the timing of the post-exercise measurements in this study. All supplements were prepared by an independent member of the team in order to maintain the double blind design of the study. To test compliance with the supplementation regime, participants were required to complete a daily supplementation log pertaining to the timing and ingestion of the supplement as well as any adverse effects. 

### 2.4. Supplement Analyses

Each 330 mL serving of the Ecuadorian cacao mucilage juice contained 68.0 g carbohydrate, which provided 277 kCal and 154 mg polyphenols (Atlas Bioscience Inc, Tucson, AZ, USA, modified Folin-Denis reagent method) including 8 mg epicatechin (Atlas Bioscience Inc, High performance liquid chromatography, HPLC, Tucson, AZ, USA), 43 mg catechins (Atlas Bioscience Inc, HPLC, Tucson, AZ, USA), 23 mg flavanols (Atlas Bioscience Inc, HPLC/MS, Tucson, AZ, USA), and 12 mg proanthocyanidins (Atlas Bioscience Inc, HPLC, Tucson, AZ, USA). The placebo was an isoenergetic synthetically derived fruit-flavored drink that was designed to have similar consistency, color, and carbohydrate content (sugar and volume-matched) but without the phytochemical content of the cacao juice (Dextro Energy, GmbH, Meerbusch, Deutschland). The placebo consisted of water, dextrose (25%), inverted-sugar syrup (22%), acid (citric acid), flavoring, and coloring (E 150 c). Importantly, this investigation ensured the PL supplement contained equal carbohydrate (CHO) content since acute CHO ingestion has previously been reported to influence the appearance of IL-6 in plasma following strenuous exercise [36,37]. 

### 2.5. Blood Analysis

At each time point, fasting venous blood samples were collected into 10 mL vacutainers with one containing a gel for serum separation and one heparin coated. Heparinized samples were immediately centrifuged at 4500 rpm for 10 min at 4 °C before having the plasma removed and stored in eppendorfs. Samples containing no anti-coagulant were left at room temperature for one hour and then centrifuged at 4500 rpm for 15 min at 4 °C. Eppendorfs were then immediately stored at −80 °C and later analyzed for serum creatine kinase (marker of muscle damage), serum protein carbonyls (marker of oxidative damage), and a C–reactive protein (hsCRP) and interleukin 6 (IL-6, markers of inflammation) by using a high sensitivity enzyme-linked immunosorbent assay (R&D Systems^®^, Minneapolis, MN, USA). Intra-sample CVs ranged from 0.7% to 6.8%. Serum CK and hsCRP concentrations were determined using colorimetric and turbidometric assays on a Roche/Hitachi Cobas c 702 analyzer and using commercially available reagents (hsCRP: PZ Cormay, Lublin, Poland, inter-assay CV 3.34%, CK: Siemens Medical Solutions Diagnostics Limited, Berks, UK, inter-assay CV 3%). Intra-sample CVs ranged from 0.6% to 4.0%. Protein carbonyls (Oxiselect™, Cell Biolabs Inc, San Diego, CA, USA, inter-assay CV 8%) were quantified using commercially available ELISA kits. Changes over time were quantified as both percentage and absolute differences from baseline values.

### 2.6. Data Analyses

The maximal isometric force was calculated as the average force over 1-s periods during the force plateau of each MVC contraction. The highest of three MVCs was accepted as the MVC force. The MVC force at each time point was normalized to baseline values for the respective trial. In addition, the highest of three CMJs was accepted as the CMJ height and normalized as a percentage of baseline values for the respective trial. Group mean total caloric and relative macronutrient intake for each trial was calculated via a nutrition analysis software system (V4.0, Nutritics LTD, Dublin, Ireland). 

### 2.7. Statistical Analyses

To determine whether there were any effects of time or trial on recovery from muscle damage, data were analyzed using two way repeated measures ANOVA (trial, 2 levels: CJ and PL vs. time, 4 levels; Baseline, Post, 24 h post, 48 h post) of muscle function, perceptual variables, and physiological variables. Where the ANOVA revealed a significant interaction effect, post-hoc tests were completed using a Bonferroni correction. For a calculation of the effect size, partial eta squared (η^2^) was used for omnibus tests. Cohen’s d was used to calculate the effect size for paired t-tests and post-hoc comparisons. Paired samples t-tests were used to assess differences in leg dominance and order effect on MVC and CMJ. All statistical tests were performed on both the percentage change and raw data. Mauchley’s Test of Sphericity was used to assess homogeneity of data and, where violations were present, corrections were made using the Greenhouse-Geisser adjustment. For all tests, results were considered statistically significant when *p* < 0.05. Data are presented as means ± SD unless otherwise indicated. All statistical analyses were conducted using SPSS Statistics version 24 (IBM, New York, NY, USA). The sample size (n = 10) provided 80% power to detect a 5% difference between trials (effect size of 1). These calculations were based on the recovery of MVC at 48 h post damaging exercise from published work [10] and the anticipation of a curvilinear relationship between dose and functional effects of supplementation.

## 3. Results

The mean exercise load during the intense knee extension exercise protocol was 79% of 1RM. There were no differences in total caloric intake between trials in the 48 h pre and post muscle damage (PL: 2650 ± 191 vs. CJ: 2752 ± 233, *p* > 0.05). Similarly, there were no differences in carbohydrate (PL: 305 ± 29 vs. CJ: 316 ± 29), lipid (PL: 91 ± 21 vs. CJ: 81 ± 36), or protein (PL: 103 ± 4 vs. CJ: 104 ± 7) intakes (all *p* > 0.05). There was no difference between trials in baseline knee-extension MVC (CJ: 296 ± 58 vs. PL: 286 ± 47 N.m, *p* = 0.339), 1RM (CJ: 90 ± 21 vs. PL: 91 ± 17 kg, *p* = 0.755) or CMJ (CJ: 53.8 ± 7.1 vs. PL: 53.7 ± 7.0 cm, *p* = 0.594). Performance, functional, and perceptual variables were not influenced by leg dominance or order of trials (all *p* > 0.05).

### 3.1. Muscle Function and Muscle Soreness

**Figure 2:**
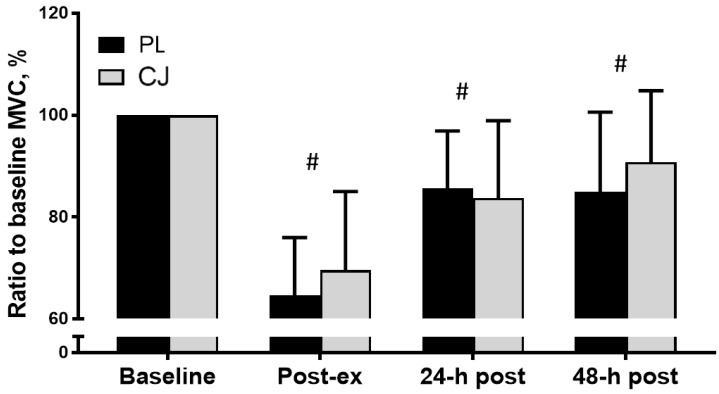
MVC force normalized to baseline values following 10 sets of 10 single knee extensions at ~80% 1RM with ingestion of placebo (PL, black bars) or Cacao juice (CJ, grey bars). There was a main effect of time in both trials (*p* < 0.05) but no interaction effect (*p* > 0.05). The numbers are significantly different from the baseline.

Across all trials, knee extension MVC decreased to a mean of 67% of baseline levels following the intensive exercise protocol (PL: 64.7 ± 11.3 vs. CJ: 69.6 ± 15.4%, d = 0.368) demonstrating a significant main effect of time (F_(1, 3)_ = 30.488, *p* < 0.0001, η^2^ = 0.772). However, there was no main effect for trial (*p* = 0.195, η^2^ = 0.179) or an interaction effect (*p* = 0.445, η^2^ = 0.093) with levels returning to 83.7 ± 15.2% in the CJ trial (vs. 85.7 ± 11.2%, placebo trial, d = 0.052) after 24 h post recovery and to 90.8 ± 14.0% in the CJ trial (vs 85.1 ± 15.6%, placebo trial, d = 0.391) after 48 h post recovery, respectively (Figure 2). A similar pattern was observed for non-normalized MVC force data with both absolute change (trial by time interaction, *p* = 0.445, η^2^ = 0.093) and absolute MVC force (main trial effect, *p* = 0.200, η^2^ = 0.179, Table 1) not different between trials. 

**Figure 3:**
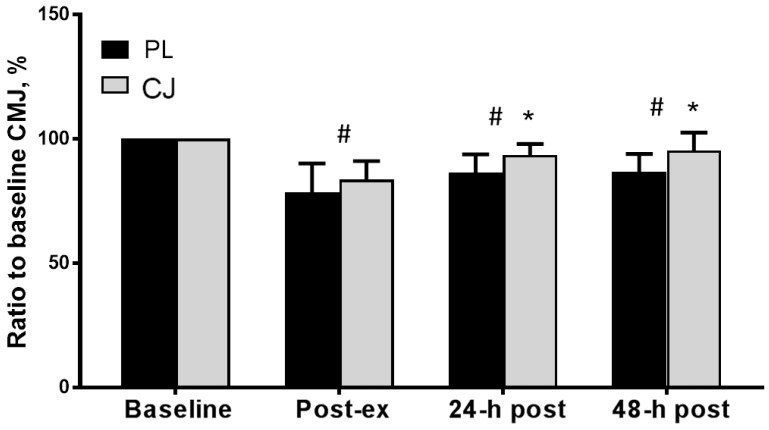
CMJ height normalized to baseline values following 10 sets of 10 single knee extensions at ~80% 1RM with ingestion of placebo (PL, black bars) or Cacao juice (CJ, grey bars). There was a main effect of time in both trials (*p* < 0.05) and a significant interaction effect (*p* < 0.05) with an enhanced MVC force recovery with CJ compared to PL. * Significantly different from PL. # Significantly different from baseline (*p* < 0.05).

**Figure 4:**
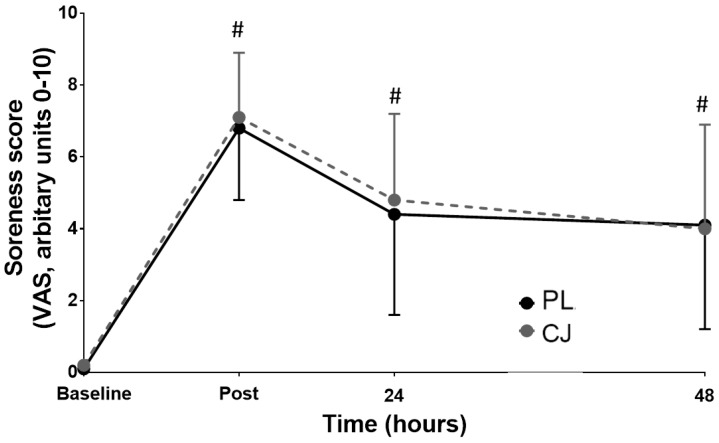
Subjective muscle soreness scores (VAS) following 10 sets of 10 single knee extensions at ~80% 1RM with ingestion of placebo (PL, black circles, solid line) or Cacao juice (CJ, grey circles, dashed line) (0–10 scale). Values are means ± SD. # Significantly different from baseline (*p* < 0.05).

There was also a significant main effect of time on CMJ (F_(1, 3)_ = 30.962, *p* < 0.0001, η^2^ = 0.775). However, CMJ recovery was significantly faster following CJ consumption than PL, which demonstrates a significant main effect of trial (*p* = 0.002, η^2^ = 0.685) and interaction effect (*p* = 0.013, η^2^ = 0.324) with levels returning to 83.5 ± 7.4% of baseline (i.e., 100%) immediately post-exercise (vs. 78.5 ± 11.5%, placebo trial, d = 0.529), 93.4 ± 4.5% (vs 86.3 ± 7.4%, placebo trial, d = 1.186) after 24 h recovery and to 95.2 ± 7.2% (vs 86.7 ± 7.3%, placebo trial, d = 1.196) after 48 h recovery relative to baseline, respectively (Figure 3). There was a significant main effect of time on VAS (*p* < 0.0001, η^2^ = 0.740), which indicates the development of muscle soreness. However, there was no significant difference between trials (main trial effect, *p* = 0.597, η^2^ = 0.032) or interaction effect (*p* = 0.749, η^2^ = 0.043, Table 1, Figure 4).

### 3.2. Blood Markers

There was a significant increase in serum CK activity after the exercise protocol by 12%–41% (main effect of time, *p* = 0.007, η^2^ = 0.357, Table 2) even though no trial (*p* = 0.274, η^2^ = 0.131) or interaction effects (*p* = 0.294, η^2^ = 0.126) were found. The percentage increase in serum CK activity from baseline was not statistically different between trials after 24 h (102.3 ± 76.0 vs. 93.5 ± 140.5%, d = 0.081) and 48 h (196.4 ± 280.3 vs. 102.2 ± 123.3%, *p* > 0.05, d = 0.467) recovery from the exercise protocol for CJ and PL, respectively. However, the absolute increase in serum CK activity tended to be higher during the CJ than the placebo trial (Table 2). 

No main effects of time (*p* = 0.211, η^2^ = 0.152, Table 2), trial (*p* = 0.631, 251 η^2^ = 0.027, Table 2), or time by trial interaction (*p* = 0.731, η^2^ = 0.046) existed for IL-6 (Table 2). In addition, there was no main effect of time (*p* = 0.238, η^2^ = 0.143), trial (*p* = 0.855, η^2^ = 0.004) or interaction (*p* = 0.494, η^2^ = 0.084, Table 2) on hsCRP. Serum protein carbonyl content was unchanged after the exercise protocol (*p* = 0.987, η^2^ = 0.005, Table 2) and there was no difference between CJ and PL (*p* = 0.984, η^2^ = 0.001) or an interaction effect (*p* = 0.574, η^2^ = 0.070). 

## 4. Discussion

The main finding of this study was that consumption of 330 mL CJ 7 days prior to and 2 days after intense knee extension exercise had no effect on the recovery of MVC force, muscle soreness (assessed via VAS), suppression of muscle damage (CK), inflammation (hsCRP or IL-6), or oxidative stress (PC). However, the ingestion of CJ was associated with improved recovery of explosive power (as inferred from CMJ) compared to PL. 

### 4.1. Functional Tests

This is the first study to suggest that supplementing with CJ, containing proanthocyanidins and flavanols such as catechins, may enhance muscle function following muscle damaging exercise. While CJ supplementation had no statistical effect on MVC recovery, recovery of CMJ performance was enhanced at 24 and 48 h, and the magnitude of this effect (~6%) was significantly greater than the CV for assessment of CMJ (1.4%). In contrast, MVC recovery was not significantly enhanced after CJ. Previous studies have found that fruit-derived polyphenol supplementation (~1000 mg) enhances recovery of isometric muscle strength [10,11,12,13,14,20]. However, in this study, CJ provided a different blend of polyphenols (flavanols and proanthocyanidins versus anthocyanins) and a lower dose of polyphenol (100 versus 1000 mg/day), which might explain the discrepancy in findings.

### 4.2. Muscle Damage, Oxidative Stress, and Inflammation

The significant rise in serum CK in the current study is indicative of damage to the sarcolemma, which allows efflux of the large CK molecule into the extracellular fluid compartment. Despite the improvement in recovery of CMJ, CJ did not appear to exert effects on CK activity. However, serum CK concentration is a blunt tool for assessing the degree of muscle damage and this finding concurs with Bowtell et al. (2011) and Howatson et al. (2010) where, despite the improved functional recovery following muscle damaging exercise and the consumption of Montmorency cherry juice, the increases in CK were also not different between trials [10,11]. No reduction in soreness after polyphenol supplementation was also evident. However, the existing literature is conflicting with some studies finding that polyphenol supplementation attenuated delayed onset of muscle soreness [9,14,38,39] while others found no effect [10,11,16,20] most likely due to the subjectivity of the measure. 

In contrast to our hypothesis, CJ did not attenuate oxidative damage to serum proteins or inflammatory markers. However, the exercise protocol failed to elevate plasma protein carbonyls and, in this circumstance, it is not possible to observe any suppression of oxidative damage. There is considerable variation across studies in the oxidative damage response to resistance exercise with some groups finding evidence of oxidative damage [10,40,41,42,43] and others not finding any such evidence [44,45]. This variation may, in part, be explained by differences in the exercise mode (i.e., single versus repeated days exercise), the training status of participants, and the use of a variety of assays to detect oxidative damage. It is also possible that the polyphenol dose provided in our study was not sufficient to induce anti-oxidant or anti-inflammatory effects. However, anti-inflammatory effects of polyphenol supplementation have been demonstrated in studies that involved whole body exercise including a larger proportion of muscle mass [9,12,13].

### 4.3. Experimental Considerations

Previous research has demonstrated that peak systemic anthocyanin bioavailability is achieved 1 to 2 h post ingestion [35,37]. In this study, participants ingested the supplement immediately after the intense knee extension exercise protocol and 60 min prior to assessment of muscle function 24 h and 48 h post exercise. This may have important implications for the potential of antioxidants to improve acute functional recovery from exercise. Previous studies [12] have timed the antioxidant dose to 60–120 min prior to exercise such that peak systemic anthocyanin concentration will occur during the exercise trial as well as 24 h and 48 h post-exercise. However, it should also be noted that a lower higher polyphenol dose was provided in the present study, which may have contributed to the lack of effects on functional recovery, oxidative stress, and inflammation. 

Polyphenols have also been suggested to influence vascular function [46]. In the current study, it is, therefore, plausible that the polyphenol content of CJ may have influenced endothelial function by allowing for improved blood flow and contributing to improved muscle function without influencing other blood markers. However, since we did not measure vascular function, further work is needed to identify the precise mechanism by which CJ might accelerate recovery despite an improvement in CMJ recovery.

It is also of interest to note that CJ positively impacted functional recovery of an explosive power-predominant exercise (i.e., CMJ) without enhancing recovery of a maximum strength exercise (i.e., MVC). An important feature in CMJ is that, apart from an MVC, speed is a key element. While we currently have no mechanistic data to support such speculation, it is possible that the polyphenol blend of CJ could account for the improvements only (or more pronounced improvements) in CMJ such that vascular effects of polyphenols contained within CJ may play a more pronounced role in the observed results (i.e., enhanced recovery of CMJ vs. MVC or power vs. strength) as the energetics and physiological mechanisms that underpin these types of contractions (i.e., dynamic vs. isometric) are considerably different [47]. However, it is also possible that the measurement of MVC in this study was not sensitive enough to detect small differences in recovery. Given that we observed functional changes with no mechanistic explanation, the effect we observed on CMJ may purely be coincidental such that the low dose of polyphenols may not have had any effect on recovery from the intense knee extension exercise protocol. Therefore, the effect of CJ on CMJ recovery warrants further investigation since it is currently unsupported by mechanistic data and other measurements of functional and perceptual recovery.

## 5. Conclusions

Cacao juice supplementation for seven days prior to and two days after intensive single leg knee extensor exercise did not improve maximal isometric strength recovery or plasma biomarkers of inflammation or oxidative damage but did enhance recovery of explosive power. The lower dose (100 versus 1200 mg polyphenols) and different blend (flavanols and proanthocyanidins versus anthocyanins) of polyphenols may account for the weaker effects on recovery than observed in other polyphenol supplementation studies. The observed augmentation of recovery of muscle function is particularly relevant for situations with constrained recovery duration such as the tournament setting. 

## Figures and Tables

**Table 1:** Group mean ± SD for measures of non-normalized knee-extensor MVC force, CMJ and VAS following 10 sets of 10 single knee extensions at ~80% 1RM with ingestion of PL or CJ.

Measures	Baseline	Post	24 h Post	48 h Post
MVC (N.m)				
PL	286 ± 47	184 ± 44 *	245 ± 53 *	243 ± 66 *
CJ	296 ± 58	204 ± 56 *	248 ± 64 *	269 ± 65 *
CMJ (cm)				
PL	53.8 ± 7.1	42.6 ± 8.1 *	46.5 ± 8.1 *	46.8 ± 8.7
CJ	53.7 ± 7.0	45.1 ± 7.9 *^,#^	50.3 ± 8.1 *^,#^	51.4 ± 9.6 *^,#^
VAS (au)				
PL	0.1 ± 0.1	6.8 ± 2.0 *	4.4 ± 2.8 *	4.1 ± 2.9 *
CJ	0.2 ± 0.1	7.1 ± 1.8 *	4.8 ± 2.4 *	4.0 ± 2.9 *

PL, placebo. CJ, Cacao juice. MVC, maximal voluntary contraction. CMJ, counter movement jump. VAS, visual analogue scale. N.m, Newton meters. au, arbitrary units. * significantly different from baseline, ^#^ significantly different from placebo, *p* < 0.05.

**Table 2:** Group mean ± SD for blood makers serum creatine kinase (marker of muscle damage), serum protein carbonyls (marker of oxidative damage), C-reactive protein, and interleukin 6 (markers of inflammation) following 10 sets of 10 single knee extensions at ~80% 1RM with ingestion of PL or CJ.

	Baseline	Post	24 h Post	48 h Post
CK (mg/L)				
PL	262 ± 284	287 ± 312 *	389 ± 424 *	402 ± 429 *
CJ	303 ± 289	331 ± 305 *	389 ± 231 *	510 ± 370 *
PC (nmol/mg)				
PL	1.72 ± 1.76	1.84 ± 1.83	2.03 ± 2.14	1.69 ± 1.72
CJ	1.77 ± 1.76	1.74 ± 1.72	1.74 ± 1.76	1.93 ± 1.92
hsCRP (mg/L)				
PL	0.72 ± 0.71	0.77 ± 0.76	0.66 ± 0.68	0.50 ± 0.50
CJ	0.71 ± 0.77	0.69 ± 0.72	0.73 ± 0.61	0.67 ± 0.46
IL-6 (pg/mL)				
PL	1.37 ± 1.43	1.62 ± 1.58	1.15 ± 1.07	1.50 ± 1.31
CJ	1.25 ± 1.37	1.70 ± 1.69	1.44 ± 1.34	1.82 ± 1.81

PL, placebo. CJ, Cacao juice. CK, creatine kinase. PC, protein carbonyls. hsCRP, C-reactive protein. IL-6, interleukin-6. * significantly different from baseline, *p* < 0.05.
